# A Cross-Sectional Study of the Nutritional Quality of New South Wales High School Student Food and Drink Purchases Made via an Online Canteen Ordering System

**DOI:** 10.3390/nu13124327

**Published:** 2021-11-30

**Authors:** Tara Clinton-McHarg, Tessa Delaney, Hannah Lamont, Christophe Lecathelinais, Sze Lin Yoong, Luke Wolfenden, Rachel Sutherland, Rebecca Wyse

**Affiliations:** 1 Hunter New England Population Health, Wallsend, NSW 2287, Australia; tara.clintonmcharg@health.nsw.gov.au (T.C.-M.); tessa.delaney@health.nsw.gov.au (T.D.); hannah.lamont@health.nsw.gov.au (H.L.); christophe.lecathelinais@health.nsw.gov.au (C.L.); syoong@swin.edu.au (S.L.Y.); luke.wolfenden@health.nsw.gov.au (L.W.); rachel.sutherland@health.nsw.gov.au (R.S.); 2 School of Medicine and Public Health, The University of Newcastle, Callaghan, NSW 2308, Australia; 3 Hunter Medical Research Institute, Newcastle, NSW 2300, Australia; 4 Priority Research Centre for Health Behavior, The University of Newcastle, Callaghan, NSW 2308, Australia; 5 School of Health Sciences, Swinburne University of Technology, Hawthorn, VIC 3122, Australia

**Keywords:** canteen, online systems, high school, public health nutrition, purchasing behaviour

## Abstract

Unhealthy dietary patterns in adolescence are associated with an increased risk of future chronic disease. This study aimed to assess online canteen lunch purchases made by high school students to identify: (1) the nutrient composition of purchases (energy, saturated fat, sugar, sodium, percent energy from saturated fat and total sugar); (2) the proportion of items classified as healthier (‘Everyday’) and less healthy (‘Occasional’ or ‘Should not be sold’) according to the New South Wales Healthy Canteen Strategy; (3) the frequency of purchases by product type (e.g., salty snacks), their classification and nutrient composition; and (4) associations between student characteristics and the nutrient composition and classification of purchases. The average order contained 2075 kJ of energy, 6.4 g of saturated fat, 18.4 g of sugar and 795 mg of sodium. Less healthy (‘Occasional’ and ‘Should not be sold’) items combined accounted for 56% of purchases. The most frequently purchased products were burgers and crumbed/coated foods. Students in higher grades purchased a significantly higher mean percent of ‘Everyday’ items, compared to students in grades 7 or 8. The majority of high school student purchases were less healthy (‘Occasional’ or ‘Should not be sold’) items, warranting further investigation of factors influencing online canteen purchasing behaviour in this setting.

## 1. Introduction

Poor nutritional intake, including low consumption of wholegrains and fruit and the overconsumption of sodium, fat and sugar, is associated with a high disease burden and increased risk of developing cardiovascular disease, cancer and obesity [1,2,3]. A number of longitudinal studies have shown that poor nutritional intake in adults is often a continuation of unhealthy dietary behaviours established in childhood and adolescence [4,5]. A high intake of energy-dense and nutrient-poor foods are known to contribute to overweight and obesity in childhood and adolescence [6]. Obesity in children and adolescents is recognised as having reached epidemic levels in many Organisation for Economic Co-operation and Development (OECD) countries [7,8]. For example, in the United States in 2016, 18.4% of children (6–11 years) and 20.6% of adolescents (12–19 years) were obese [9]. In Australia, the most recent national data reported that 25% of 5–17 year olds were either overweight or obese [10]. Therefore, it is critical to investigate the environments in which children and adolescents are forming their dietary behaviours, so that a better understanding of the types of foods and beverages consumed can be gained, and context-specific interventions to improve dietary intake can be implemented.

The school food environment plays an important role in the overall diet of adolescents (11–19 years), with students spending approximately 30 h per week at school and consuming a large amount of their dietary intake while in this setting [11]. While many countries provide school meal programmes or cafeteria-style provision of student lunches [12], other countries such as Norway, the Netherlands, Malaysia, New Zealand and Australia [13,14,15,16,17] have a school canteen or tuck-shop, which students have the option of using to purchase food and drinks in addition to, or instead of, bringing food and drinks from home. The nutritional quality of foods and drinks purchased by high school students at school canteens is often perceived as being less healthy than lunch brought from home. However, while the availability of items on canteen menus is often reported, only a few studies internationally have investigated the types of products actually purchased by secondary school students at canteens [13,17,18,19], and no studies have reported data on the nutrient composition of those products.

The lack of information regarding the nutritional quality of products purchased by students from high school canteens is concerning given the contribution they can potentially make to the adolescent diet. For example, data from the 2015 New South Wales Physical Activity and Nutrition Survey (SPANS) showed that almost one-third of adolescents frequently purchased lunch from their school canteen (≥2 times per week) [20]. Further, the SPANS data found that adolescents who frequently purchased their lunch from the canteen (≥2 times per week) were significantly more likely to have a waist-to-height ratio indicating high cardiometabolic risk, compared to adolescents who purchased their lunch from the canteen less frequently (≤1 time per week) [20]. These data highlight the need for a comprehensive assessment of food and beverage items being purchased from high school canteens, as well as which students are purchasing them, to gain a better understanding of dietary risks and to inform interventions to encourage healthy food and beverage choices for canteen users.

Despite being recognised as an important evidence gap [20], no quantitative studies have been conducted to date that describe the nutritional quality or types of foods and beverages being purchased from high school canteens in Australia. In a 2015 systematic review of studies examining the use of Australian school canteens, only five studies investigated canteen use and product availability, and only two of these included high school canteens [11,16]. Neither of these studies were based on objectively collected purchase data. One of the studies was qualitative [16], while the other study used 24 h recall data to estimate the average energy intake of foods consumed while at school, compared to the average energy intake outside of school hours [11]. The latter study found that canteen users consumed approximately 200 kJ more energy while at school and were significantly more likely to obtain energy from fast food, packaged snacks, desserts, chocolate and confectionary and milk [11]. However, the data for canteen users were not separated into foods and drinks brought from home and those purchased at the canteen. Neither of these previous studies provided data on the types of products being most frequently purchased at the canteen or the nutritional composition of foods and beverages purchased.

Given the importance of the high school setting to the diet of adolescents and the lack of studies that have explored canteen purchases, the current study aimed to identify the types of food and drink products being purchased by high school students using online canteens and assess their nutritional profile to better understand the contribution of canteen-purchased food to the adolescent diet. Specifically, the study aimed to (1) assess the nutrient composition of student orders, including the average energy (kJ), saturated fat (g), total sugar (g), sodium (mg), percent of energy from saturated fat and percent of energy from total sugar; (2) identify the overall proportion of all items purchased that were classified as healthier (‘Everyday’) or less healthy (‘Occasional’ or ‘Should not be sold’); (3) identify the most frequently purchased product types (e.g., burgers, salty snacks, fruit, etc.), and their classification and nutrient composition; and (4) examine associations between student characteristics (school grade and frequency of canteen purchasing) and the mean proportion of healthier (‘Everyday’) items purchased, as well as the nutrient composition of orders.

## 2. Materials and Methods

### 2.1. Design

This cross-sectional study was carried out using canteen purchasing data from nine schools catering for high school students (Grades 7–12, aged 11–19 years) in New South Wales (NSW) Australia over a 10-week period (October to December 2020). The state of NSW is geographically diverse with metropolitan, regional, rural and remote areas. In 2020, it had 469 government high schools (including 68 combined primary/secondary schools) attended by 312,000 high school students, and 394 non-government high schools (including 254 combined primary/secondary schools) attended by 220,000 high school students [21]. Ethics approval for the study was granted by the University of Newcastle Human Research Ethics Committee (H-2017-0402), the New South Wales Department of Education (SERAP 2018065) and the appropriate committees for non-government schools.

### 2.2. Participants

Schools: Eligible schools could be government (public state funded) or non-government (e.g., catholic, independent) schools in NSW that enrolled high school students. This included schools that enrolled high school students only (Grades 7–12), as well as combined schools enrolling students from Kindergarten to Grade 12, as data for high school students could be extracted separately. The schools needed to be using an online canteen ordering system provided by ‘Flexischools’, the leading online canteen system provider in Australia used by more than 1400 primary and high schools [22]. Online canteen ordering is increasingly common within Australia, as in other food settings. Users login (usually via a mobile phone application) to access the school’s canteen menu and are able to select food and drink items and pay for them online. Analysing data collected via online canteen ordering systems provides an efficient way of investigating purchases, as the system automatically records the orders of all users in a centralised location.

Schools were ineligible to participate if they did not use an online canteen ordering system provided by ‘Flexischools’. Schools that catered exclusively for students with special needs, hospitalised children or juvenile justice students were also excluded due to the differences in food provision that occurs in these settings.

### 2.3. Participants

Students: Student purchasing data were included in the study if the student was enrolled at a participating high school, was in Grade 7–12 (i.e., aged between 11–19 years) and had made at least one online canteen purchase during the 10-week study period (Term 4, 2020). Term 4 is the last quarter of the Australian school year, which runs from October to December. Data were included if the student’s online order was for a lunch break and included at least one food or beverage item. Non-food and drink items were excluded (e.g., straws, cutlery, bags and shirts) within an order. Items within an order were also excluded if it they did not appear on the Term 4 menu (e.g., a one-off menu special) as the item was not deemed to be representative of a usual purchase. An ‘order’ was excluded if 15 or more of the same items were purchased in the one order, as consensus by dietitians experienced in school canteen research was that these types of purchases (e.g., 15 slushies) were unlikely to be for an individual student. The threshold of 15 items was selected as analysis of previous data sets in primary schools indicated that some smaller items (e.g., chicken nuggets) are commonly sold in high numbers (e.g., 12 pieces).

### 2.4. Sample

The recruitment of schools for this study occurred as part of a larger randomised controlled trial that was being conducted by the research team. Details of the trial can be found at Open Science Framework (https://osf.io/h8zfr—accessed on 15 July 2021). A list of all NSW schools enrolling high school students who were currently using the Flexischools online canteen system was provided to the research team by Flexischools. The 134 schools identified on the list were mailed an information pack and invited to participate in the randomised controlled trial between October 2020 and January 2021. The information pack included a letter to the school principal, a letter to the canteen manager and a consent form for the principal to sign and return. Approximately 1–2 weeks following the mailed information pack, a member of the research team contacted the school canteen manager by phone to provide an overview of the study, confirm the school’s interest in participating and answer any questions related to the study. Phone calls were made to schools in a random order. Recruitment continued until ten school principals had consented for their school to take part in the trial. One school was deemed to be ineligible to participate after providing consent, leaving a sample of nine high schools for inclusion in the randomised controlled trial and this current cross-sectional study.

### 2.5. Measures

#### 2.5.1. Food and Beverage Nutrient Composition

Purchasing data collected from participating schools for 10 consecutive weeks of Term 4 2020 were provided to the research team by Flexischools. An experienced research dietitian used a school canteen menu assessment procedure, applied in previous studies [23,24], to identify the nutrient composition (per serve) for every item on each school’s Term 4 menu. The nutrients assessed were energy (kJ), saturated fat (g), total sugar (g), sodium (mg), percent of energy from saturated fat and percent of energy from total sugar. These nutrients were chosen due to their established associations with obesity and chronic disease [1,2,3]. The mean percent of energy derived from saturated fat (37 kJ/g) and sugar (17 kJ/g) were based on internationally accepted conversion factors [25]. The procedure used to assess menu items was as follows: (1) The research dietitian telephoned each school’s canteen manager to confirm the brand, product name, serve size and flavour of commercially available menu items and to obtain a detailed list of ingredients and serving size for freshly prepared (canteen-made) items. (2) Using the information provided by the canteen manager, the dietitian attempted to match the item to an internal database of over 2000 commonly stocked canteen items (developed over the last 10 years by the broader study team) to identify the nutrient information for the product. (3) If the product was not included in the database, the dietitian would search for the product’s nutritional information using the FoodSwitch website or app [26]; perform online searches to locate the product’s nutrition information panel (NIP); or if the NIP could not be found on the internet, the dietitian would contact the product manufacturer or supplier to request the nutrition information. (4) If the item was canteen made, the ingredients were entered into the FoodWorks Version 9 nutrition analyses programme so that the nutritional profile of the item could be generated [27].

#### 2.5.2. Food and Beverage Classification

Items on each school’s Term 4 menu were also classified by the research dietitian as ‘Everyday’, ‘Occasional’ or ‘Should not be sold’ according to the NSW Healthy School Canteen Strategy: food and drink criteria [28]. The NSW Healthy Canteen Strategy is based on the Australian Dietary Guidelines [29] and was developed by a working group, comprising representatives from the NSW Department of Education, NSW Ministry of Health, the Association of Independent Schools of NSW and Catholic Schools NSW [28]. Classifications are based on a range of factors that can include the product’s nutrient profile, Health Star Rating, serve size and source of preparation (i.e., commercial product or canteen made). The NSW Healthy Canteen Strategy recommends that 75% or more of the items on the canteen menu should be ‘Everyday’ items and no more than 25% of items on the canteen menu should be ‘Occasional’ items [28]. Items that ‘Should not be sold’ in school canteens according the strategy include all sugar-sweetened drinks; fruit drinks, slushies or ice-blocks with less than 99% juice; sugar-sweetened jelly; most confectionary; foods with added confectionary (e.g., choc-chip biscuits, cakes with icing); and items that exceed the maximum portion limit or have a Health Star Rating lower than that needed to be classified as ‘Everyday’ and ‘Occasional’ [28]. A detailed description of the product types that are classified as ‘Everyday’ and ‘Occasional’ can be seen in Table S1. To classify items as ‘Everyday’, ‘Occasional’ or ‘Should not be sold’ (SNBS), the research dietitian used the same Steps 1–3 outlined above, except that, at Step 3, the Healthy Food Finder website was used to check food and drink classifications in addition to the Foodswitch app [30]. FoodWorks (Step 4 above) was not used to classify canteen-made products, instead the dietitian assessed the portion size and ingredients of canteen-made products against the NSW Healthy Canteen Strategy guidelines to determine classification.

#### 2.5.3. School Characteristics

Characteristics including the school sector (government or non-government), school type (high school or combined school), number of students enrolled and school postcode were obtained from the 2020 data published on the government ‘MySchool’ website [31]. The MySchool website publishes publicly available demographic details of all government and non-government schools. For combined schools, the school registrars were emailed to provide enrolment numbers for high school students only, as these data are not available on the MySchool website. Details regarding canteen operation (P&C/School run or external provider) were accessed from school websites and opening periods of canteens were captured as part of the Flexischools data.

#### 2.5.4. Student Characteristics

The grade levels of students (grade 7–8, grade 9–10, grade 11–12) and frequency of online canteen purchasing (<1 order per week on average or ≥1 order per week on average) were identified from the Flexischools data set. The average number of items purchased per lunch order and average cost per order were also calculated using the Flexischools data.

### 2.6. Data Analyses

Analyses were conducted using SAS version 9.3 (SAS Institute Inc., Cary, NC, USA). Descriptive statistics (frequencies, proportions, means, standard deviations) were used to describe the characteristics of the schools and canteens participating in the study, and student characteristics (grade, frequency of online canteen purchasing, average number of items purchased per order, average cost per order). The school postcode was used to determine the geographic location of the school (major city or inner regional area) according to the Australian Statistical Geography Standard (ASGS) Edition 2 [32]. The school postcode was also used to determine each school’s socio-economic region (most advantaged or least advantaged) according to the Socio-Economic Indexes for Areas (SEIFAs) [33]. Schools were dichotomised according to the New South Wales median SEIFA, with schools above the median being classified as most advantaged, and schools below the median being classified as least advantaged.

A visual examination of histograms confirmed that the distribution of data was nonparametric. Therefore, medians, interquartile ranges and minimum and maximum values were used to decribe the nutrient composition (energy, saturated fat, total sugar, sodium, percent of energy from saturated fat, percent of energy from total sugar) of student lunch purchases in addition to means. A ‘purchase’ could contain more than one item, therefore nutritional information for all items in the order were added together so that the overall nutrient composition of the purchase became the unit of analyses. Potential outliers for purchase data (data beyond 1.5 times the interquartile range) were independently checked by two dietitians prior to analyses; however, there was agreement that all of the identified purchases met the inclusion criteria, and they were included in the analyses.

All analyses conducted to determine the classification of food and drinks considered the individual item (not overall purchase) as the unit of analyses. The classification of the items that students purchased were described using frequencies and proportions.

The top 10 most frequently purchased product types were identified using frequencies, and the average proportion of total items purchased that a product represented across all nine schools was reported using means, standard deviations, and ranges (minimum to maximum proportions). Means and standard deviations were used to describe the average nutrient composition of these product types (energy, saturated fat, total sugar, sodium, percent of energy from saturated fat, percent of energy from total sugar).

Linear mixed-effect regression models were used to identify any associations between student characteristics and the mean nutrient composition and mean percent of ‘Everyday’ items ordered by students at lunch. The continuous outcome variables in the models were energy (kJ) per order, percent of energy from saturated fat per order, percent of energy from total sugar per order and percent of ‘Everyday’ items. Student grade (grade 7–8, grade 9–10, grade 11–12) and frequency of canteen purchasing (<1 order per week on average or ≥1 order per week on average) were included as the predictor variables. All models included a random intercept for school to account for clustering. Additionally, the nutrient composition models included a random intercept for the user to account for repeated measures. All models also included a fixed effect for school socio-economic region (most advantaged, least advantaged) and school sector (government, non-government). The statistical significance for all models was *p* < 0.05.

## 3. Results

Descriptive characteristics of the nine schools who participated in the study can be seen in Table 1. All participating schools had canteens that operated five days per week. Two of the schools were boys-only high schools.

Compared to the characteristics of schools across the state of NSW, our sample had more non-government schools (67%, compared to 46% in NSW) and combined schools (56%, compared to 37% in NSW) [21]. The majority of the schools in the sample were located in major cities (67%, compared to 55% in NSW) and socio-economically advantaged regions. Eighty-nine percent of canteens were internally run by the school and/or the schools’ parents and citizens (P&C) group.

Table 2 presents the characteristics of students using online canteen ordering for lunch breaks. There were an estimated 5981 secondary students enrolled at the 9 participating schools in 2020. Of these, 1402 students (23%) made at least 1 online lunch purchase during the 10-week study period. Online canteen purchasing was primarily used by younger grades (grades 7–10), with grades 7–8 responsible for 57% of purchases. Just over one quarter of students (28%) used the online canteen to order lunch more than once per week on average. The average number of items purchased by students per order was less than 2 (1.7), with an average cost of AUD 6.05 per order.

The nutrient composition of orders purchased by online canteen users for lunch breaks can be seen in Table 3. The average lunch order contained 2075 kJ of energy, 6.4 g of saturated fat, 18.4 g of sugar and 795 mg of sodium. The average percent of energy from saturated fat and total sugar per order was 11% and 13%, respectively.

The total number of items purchased across the 10-week period was 20,032. Of these, healthier items classified as ‘Everyday’ accounted for 44% of items purchases (n = 8880), while less healthy items classified as ‘Occasional’ made up 35% of items purchased (n = 6962) and 21% were classified as ‘Should not be sold’ (n = 4190).

The 10 most frequently purchased product types over the 10-week period can be seen in Table 4. Of these, five of the product types were classified as ‘Everyday’, four were classified as ‘Occasional’, and one was classified as ‘Should not be sold’ (SNBS). Each of the top 10 product types constituted between 3.8% and 8.3% of all items purchased (average percentage across the 9 schools).

The most frequently purchased items from canteens at lunch were burgers, crumbed/coated foods and flavoured milk drinks, accounting for 22.6% of total items purchased on average across schools. However, within individual schools, the percent of items purchased ranged from 0% to 27% for crumbed/coated foods and 0% to 18% for burgers.

Of the top 10 products purchased, sandwiches/wraps/rolls with occasional fillings and burgers had the highest mean energy (1914 and 1845 kJ, respectively) and sodium (817 and 964 mg, respectively). Pies and flavoured milk drinks had the highest mean percent of energy from saturated fat (19% and 18%, respectively). Sugar-sweetened drinks had the highest mean sugar (37.1 g) and the highest mean percent of energy from sugar (96%).

Table 5 presents the results of the linear mixed-effect regressions models to examine associations between online canteen user characteristics and the nutrient composition of orders and classification of items purchased at lunch. Student grade was significantly associated with the mean proportion of ‘Everyday’ items purchased, with students in older grades purchasing a significantly higher mean proportion of ‘Everyday’ items, compared to students in grades 7 and 8.

## 4. Discussion

This cross-sectional study investigated the online canteen purchases of students from nine NSW high schools over a 10-week period. It is the first time that a detailed investigation of canteen purchasing by high school students has been conducted in this Australian state. The study found that 23% (n = 1402) of all enrolled students made at least one online lunch purchase during the 10-week study period and students in lower grades (7–8) accounted for 57% of all orders. The average number of items purchased per order was less than two (1.7), with an average cost of AUD 6.05 per order. The average lunch order contained 2075 kJ of energy, 6.4 g of saturated fat, 18.4 g of sugar and 795 mg of sodium, and the average percent of energy from saturated fat and total sugar per order was 11% and 13% respectively. Healthier ‘Everyday’ items accounted for less than half of all purchases (44%), with the majority of lunch purchases (56%) being less healthy, including 35% ‘Occasional’ and 21% ‘Should not be sold’ items. Student grade was significantly associated with the mean proportion of ‘Everyday’ items purchased, with students in older grades purchasing a significantly higher mean proportion of ‘Everyday’ items, compared to students in grades 7–8.

Burgers, crumbed/coated foods (e.g., chicken nuggets, tenders, and schnitzels, fish fingers, arancini, tempura) and flavoured milk drinks were the most common products purchased at lunch and accounted for 22.6% of total items purchased on average across schools. Amongst beverages, flavoured milk drinks, ≥99% fruit/vegetable juices, and sugar-sweetened drinks were the most frequently purchased products. The nutrition composition of items purchased ranged by product type. Of the top 10 product types, burgers contained the highest mean sodium (964 mg), pies contained the highest mean saturated fat (7.2 g) and mean percent of energy from saturated fat (19%), and sugar-sweetened drinks contained the highest mean sugar (37.1 g) and mean percent of energy from sugar (96%).

Across the 10 week period, one-fifth of all items purchased were classified as ‘Should not be sold’ according to the NSW Healthy Canteen Strategy, which means they should not be available for purchase in NSW school canteens at all. Despite this, sugar-sweetened drinks (known to be over consumed by adolescents [34]) were the third most frequently purchased beverage from school canteens, and whilst they only accounted for an average of 3.5% of purchases across all schools, they comprised up to 13% of purchases in some individual schools. These findings highlight that despite the existence of guidelines that aim to prevent the sale of such items, ‘Should not be sold’ items are still available in canteens and being purchased by students. Interventions that target both the availability of ‘Should not be sold’ items in canteens and the purchasing behaviour of high school students may therefore be warranted.

The purchase of drinks by high school students in our sample is broadly consistent with data reported in the international literature. For example, a study by Ensaff et al. of 2461 students across two large secondary schools in England identified that juices and flavoured milk were the most common beverages selected at high school canteens [35]. These findings are consistent with our study, which also identified that flavoured milks and juices were the most popular beverages purchased from online canteens.

While the overall average energy (2075 kJ), saturated fat (6.4 g) and sugar (18.4 g) contained in student canteen orders in this study does not appear to be excessive when compared to the recommended daily intake targets for adolescents [36], it is important to note that these data only capture purchases for one meal occasion (lunch). However, the overall average amount of sodium contained in student lunch orders appears to be high. To reduce risk of chronic disease, suggested daily dietary targets of sodium intake for adolescents include 400–800 mg/day for 9–13 year olds and 460–920 mg/day for 14–18 year olds [36]. The study found that student lunch purchases contained, on average, 795 mg of sodium, representing 86% of the highest target of 920 mg/day, despite being usually only one meal occasion in a student’s day [37,38]. The high proportion of sodium within student lunch purchases is unsurprising, given the top product types purchased included burgers and sandwiches/wraps/rolls with occasional fillings (e.g., processed meat, packaged crumbed and coated foods, battered or tempura foods) each with an average sodium content of over 800 mg.

Within student orders, the average number of items purchased via online canteens appears to be small (<2 items) and seems to largely consist of main meals (e.g., burgers, sandwiches, pies, savoury pastries, crumbed/coated foods). In another study of canteen purchasing in secondary schools conducted in Northern Ireland, burgers were also the most popular main meal item for students in both urban and rural areas [19]. These findings are incongruent with other the data reported in the literature for primary school canteen purchases, which suggest that snack-type foods (e.g., savoury snacks, confectionary, ice blocks, cakes and desserts) are commonly purchased by students [39]. This could be related to student’s age; however, it may also suggest that high school students are primarily ordering main meals via the online canteen system and are potentially purchasing subsidiary products (e.g., snack type food) from the canteen over the counter. It is important to highlight that online canteen purchases made at breakfast and recess breaks were not assessed as part of the current study (due to some schools not having online ordering available for these periods). However, it is known that Flexishools do provide some schools with online canteen ordering for these break periods; therefore, it is likely that additional products are being ordered at these times. Further investigation into high school canteen purchases made across all break periods and using data obtained from both online and over the counter purchases is needed to form a more complete picture of how school canteens are contributing to the dietary intake of adolescents.

Although these data represent food and beverage purchases for only one break period, the finding that 56% of items purchased were classified as less healthy (‘Occasional’ or ‘Should not be sold’) suggests that online canteen lunch orders for high school students would be an appropriate target for intervention to reduce dietary risk factors for chronic disease. Similar findings have been reported in a study of secondary school canteen purchasing behaviour from the Netherlands, which found that both boys and girls reported purchasing a higher proportion of ‘less healthy’ items compared to ‘healthier’ items from the canteen [17]. To increase the nutritional quality of food and drinks available in school settings, many government education departments around the world have developed policies to guide schools in the types of products, and relative proportions of products, that should be available [40,41]. However, the implementation of healthy canteen policies and guidelines, within high schools is often suboptimal [17,42,43]. For example, one Australian study found that, out of 53 high school canteens, only 6% of canteen menus met the criteria of having more than 75% of menu items being ‘Everyday’ items [42]. Further, only 15% of canteens did not sell sugar-sweetened drinks classified as ‘Should not be sold’ [42]. The purchase of items classified as ‘Should not be sold’ in the current study supports these findings that some high schools are not adhering to healthy canteen guidelines, and further investigation as to why guidelines are not being implemented in this setting is needed.

Online canteen ordering systems may help facilitate the delivery of interventions to encourage the purchase of healthier items. An emerging field of research suggests that behavioural interventions can be delivered through online canteen ordering systems and can impact the food being ordered for primary school children [23,24]. Future research to examine the efficacy of dietary interventions delivered via online canteen ordering systems for high school students is needed.

Finally, the investigation of the association between the nutritional composition of lunch orders and user characteristics found no significant associations with frequency of use of the online canteen (low use vs. high use) or the mean energy, percent energy from saturated fat or percent energy from sugar. These results were unexpected, as previous research by Hardy et al. found an association between the frequency of student canteen purchasing behaviour and their weight-to-height ratio [20]. However, in the SPANS study, high purchasing frequency was defined as ≥2 times per week, whereas in the current study high purchasing frequency was defined as ≥1 times per week due to the smaller number of students in the sample. Significant associations were found between the proportion of ‘Everyday’ foods purchased and student grade, with older students purchasing a high proportion of these foods, compared to younger students in grades 7 and 8. This suggests that interventions to promote healthier purchasing from school canteens may need to be tailored to suit to younger adolescents. As such, further research to identify the effect of canteen ordering interventions on high school students by grade and age is recommended.

The findings of this study should be considered with respect to its strengths and limitations. This is the first study to analyse the nutrient composition of food and beverage products purchased by a sample of New South Wales high school students’ via online canteens. Strengths include the recruitment of schools from both government and non-government sectors and across different geographical locations and socio-economic areas. The automatic collection of purchasing data has been previously validated via in-school observations in primary schools [44], and a very high agreement (98%) was observed between the items ordered online and the items the children received at their lunch break. Further, the classification of items used to describe healthy and less healthy items in the study (‘Everyday’, ‘Occasional’, ‘Should not be sold’) was in accordance with the NSW Healthy School Canteen Strategy [28], which is applicable to every high school canteen in the state.

Limitations include that the study only assessed online canteen purchasing data and did not observe over the counter purchases which may have been made by students in addition to, or instead of, online canteen purchases. The study did not collect data regarding consumption; however, previous studies have established that objectively recorded food and beverage purchasing data are a relatively accurate estimate of actual consumption [45,46]. This study also did not assess the extent to which online canteen purchases contributed to students’ overall daily intake, as this was beyond the scope of the research. It could be that students adjusted their at-home consumption based on what they consumed at school, and further research to establish this is recommended. Probability sampling was not used to select schools for inclusion in the study. The sample of schools included two boys’ high schools and a higher proportion of non-government schools compared to the proportion of non-government schools in NSW (67%, compared to 46%), which may have implications for the generalisability of these results. The majority of schools in the study sample were also from major cities and more socio-economically advantaged areas, which may limit the generalisability of findings to schools in more rural or remote locations or in areas of lower socio-economic advantage. It should be also be noted that many grade 12 students do not attend high school in Term 4, which would account for the lower number of students from older grades purchasing from the online canteen. However, this attendance pattern is typical of NSW high schools generally and is representative of the 10-week period in the study. Individual-level student data, such as sex or age, were not provided by Flexischools and could not be included in the statistical analyses. Finally, it should be noted that data collection for this study took place while the global COVID-19 pandemic was occurring. In the state of NSW, the population levels of coronavirus in November–December 2020 were very low, and all schools included in the sample were open and delivering face to face lessons to students. Nevertheless, it is possible that student canteen purchasing behaviour may have been impacted by the pandemic, and the study results should be considered within this context. Despite these limitations, the canteen purchasing data presented in this study represent a valuable contribution to the field and further our understanding of the school food environment for adolescents.

## 5. Conclusions

This collection and analysis of 11,537 online canteen lunch orders made over a 10-week period provides useful data regarding the types of products purchased by a sample of high school students in one Australian state. Findings suggest that the majority of high school student purchases were less healthy (‘Occasional’ or ‘Should not be sold’) items. Further investigation of factors influencing online canteen purchasing behaviour in this setting is warranted.

## Figures and Tables

**Table 1:** Characteristics of participating schools and canteens (N = 9).

Characteristics of Schools and Canteens	n (%)
School sector	
Government	3 (33)
Non-government	6 (67)
School type	
Combined school (grades K-12)	5 (56)
High school (grades 7–12)	4 (44)
High school enrolments	
Number of enrolments (grades 7–12) Mean (SD)	665 (309)
School socio-economic region	
Most advantaged	6 (67)
Least advantaged	3 (33)
School geographic location	
Major city	6 (67)
Inner regional area	3 (33)
Canteen operation	
P&C/School run	8 (89)
External provider	1 (11)

**Table 2:** Characteristics of online canteen users (N = 1402).

Characteristics of Online Canteen Users	n (%)
Grade	
7–8	805 (57.4)
9–10	484 (34.5)
11–12	113 (8.1)
Frequency of canteen use	
Low (<1 order per week on average)	1008 (71.9)
High (≥1 order per week on average)	394 (28.1)
	Mean (SD)
Average number of items purchased per order ^1^	1.7 (0.8)
Average cost per order ^1^	AUD 6.05 (2.22)

^1^ Based on N = 11,537 lunch orders.

**Table 3:** Nutrient composition of student online canteen orders at lunch (N = 11,537).

	Mean (SD)	Median (IQR) ^1^	Maximum ^2^
Energy (kJ)	2075 (886)	1947 (1473–2547)	10,246
Saturated fat (g)	6.4 (5.0)	4.7 (3.2–8.7)	55.6
Total sugar (g)	18.4 (19.7)	7.5 (4.0–32.0)	170.4
Sodium (mg)	795 (378)	786 (499–985)	3376
% Energy from saturated fat ^3^	10.9 (5.6)	9.9 (7.0–14.7)	35.6
% Energy from total sugar ^3^	13.4 (12.1)	8.0 (4.1–22.0)	110.0 ^4^

^1^ Interquartile range. ^2^ Minimum value always zero. ^3^ Calculated for N = 11,492 orders, could not be calculated for orders that had 0 kJ (i.e., purchased water only). ^4^ Seven items were found to have an incorrect label for either energy or sugar, which is why the maximum exceeds 100.

**Table 4:** Ten most frequently purchased items by product type and their nutritional composition.

Product Types Classified According to NSW Product Categories	Total Items	% of Items Purchased across Schools		Energy (kJ)	Saturated Fat (g)	Sugars (g)	Sodium (mg)	% Energy from Sugar	% Energy from Saturated Fat
	n	M (SD)	Range	M (SD)	M (SD)	M (SD)	M (SD)	M (SD)	M (SD)
Burgers (Everyday)	1837	8.3 (7.7)	0–18	1845 (169)	4.7 (2.3)	8.0 (2.1)	964 (118)	7.4 (1.9)	9.1 (3.7)
Crumbed/coated foods (packaged) (Occasional)	1714	7.1 (8.9)	0–27	848 (147)	1.2 (0.9)	0.7 (1.0)	360 (76)	1.5 (1.8)	5.7 (3.9)
Flavoured milk/hot chocolate/milkshakes ^1^/smoothies ^1^/breakfast drinks/drinking yoghurt (Everyday)	1457	7.2 (4.6)	1.2–15	1189 (295)	6.0 (2.3)	36.0 (7.9)	171 (52)	52 (4.4)	18 (3.9)
Sandwiches/wraps/rolls with occasional fillings ^2^ (Occasional)	1337	6.7 (5.6)	0–14	1914 (208)	3.0 (1.2)	5.7 (2.1)	817 (106)	5.1 (2.0)	5.8 (2.2)
Sandwiches/wraps/rolls (Everyday)	882	4.8 (2.6)	1.7–9.8	1325 (214)	3.0 (1.0)	4.1 (2.1)	555 (163)	5.3 (2.5)	8.4 (2.7)
≥99% fruit/vegetable juices and coconut water (Everyday)	871	3.8 (3.4)	0–9.3	439 (58)	0.3 (0.8)	22.9 (5.3)	17 (14)	88 (14)	2.1 (5.6)
Savoury pastries (Occasional)	846	4.0 (4.0)	0–9.8	1085 (223)	4.3 (1.2)	3.2 (1.2)	312 (64)	4.8 (1.4)	14 (2.9)
Sugar-sweetened drinks (SNBS)	816	3.5 (4.5)	0–13	641 (277)	0.1 (0.2)	37.1 (16.9)	57 (41)	96 (7.0)	0.6 (1.4)
Pies (Occasional)	694	3.6 (3.4)	0–8.9	1394 (43)	7.2 (0.8)	1.9 (0.5)	519 (62)	2.3 (0.7)	19 (2.0)
Water (Everyday)	639	3.8 (3.5)	0.1–9.2	0.4 (2.8)	0.0 (0.0)	0.0 (0.0)	1.5 (5.6)	0.0 (0.0)	0.0 (0.0)

^1^ No ice cream, gelato, sorbet or frozen yoghurt added. ^2^ Occasional fillings include processed meat, packaged crumbed/coated foods and battered or tempura foods.

**Table 5:** Associations between user characteristics and the average nutrient composition of orders and classification of items purchased at lunch.

	Energy (kJ) (per Order)			% of Energy from Saturated Fat (per Order)			% of Energy from Total Sugar (per Order)			% Everyday Items (Across All Items)		
	M (SD)	β (CI)	*p*	M (SD)	β (CI)	*p*	M (SD)	β (CI)	*p*	M (SD)	β (CI)	*p*
Grade												
7–8 ^1^	1989 (818)	-	0.06	11 (5.4)	-	0.79	13 (12)	-	0.30	43 (34)	-	0.01
9–10	2198 (968)	99 (5.0, 193)		11 (5.8)	0.1 (−0.4, 0.6)		15 (13)	0.5 (−0.7, 1.7)		44 (35)	4.0 (0.2, 7.9)	
11–12	2238 (946)	137 (−28, 302)		11 (6.1)	0.3 (−0.7, 1.2)		13 (12)	−1.2 (−3.3, 1.0)		48 (35)	10.0 (3.3, 17)	
Canteen use												
Low (<1 order p/w average) ^1^	2160 (903)	-	0.35	11 (5.7)	-	0.88	14 (12)	-	0.51	44 (37)	-	0.33
High (≥1 order p/w average)	2041 (876)	−42 (−138, 54)		11 (5.6)	0.04 (−0.5, 0.6)		13 (12)	−0.4 (−1.6, 0.9)		43 (30)	−1.8 (−5.8, 2.2)	

^1^ Reference variable.

## Data Availability

The data presented in this study are available from the corresponding author on reasonable request, pending ethics approval.

## Supplementary Materials

The following are available online at https://www.mdpi.com/article/10.3390/nu13124327/s1, Table S1: Classification of food and beverage products according to the NSW Healthy Schools Canteen Strategy.
