# Supplementary material for: A Cross-Sectional Study of the Nutritional Quality of New South Wales High School Student Food and Drink Purchases Made via an Online Canteen Ordering System
Source: Nutrients. 2021 Nov 30;13(12):4327. doi: 10.3390/nu13124327 (PMC8706117; doi:10.3390/nu13124327)
Supplement: Supplementary file 1 [file nutrients-13-04327-s001.zip › nutrients-1431717-supplementary.pdf]

Table S1. Classification of food and beverage products according to the NSW Healthy Schools Canteen Strategy.

EVERYDAY ITEMS	Portion[a]	OCCASIONAL ITEMS	Portion[a]
HOT/COLD FOOD – **product categories**		HOT/COLD FOOD – **product categories**	
**Sandwiches/wraps/rolls**<br>with Everyday fillings[b]	n/a	**Sandwiches/wraps/rolls**<br>with Occasional fillings[c]	Per fillings
**Sushi/rice paper rolls**<br>with Everyday fillings[b]	n/a	**Sushi/frittata/salads**<br>with Occasional fillings[c]	Per fillings
**Salads**<br>with Everyday fillings[b]	n/a	**Processed meats (excluding ham)[d]**<br>Includes bacon, devon, salami, chicken roll, frankfurts, sausages	60g
**Hot sandwiches/toasties/ cheese melts**<br>with Everyday fillings[b]	n/a	**Hot sandwiches/toasties/wraps/rolls and burgers[d]**<br>with Occasional fillings[c]	350g
**Crumbed/coated foods (freshly prepared)**<br>Canteen-made crumbed food that is baked	140g	**Crumbed/coated foods (packaged)[d]**<br>Includes nuggets, tenders, schnitzels, fish fingers, arancini, tempura	140g
**Burgers**<br>with Everyday fillings[b] and uncoated patties	350g	**Hot meals[d]**<br>with Occasional fillings[c], processed meats, crumbed/coated foods	350g
**Jacket (baked) potatoes**<br>with Everyday fillings[b]	350g	**Hot potato products[d]**<br>Includes hot chips, hash browns, wedges, gems, skins, baked not fried	100g
**Roasts/cottage pies/fish pies**<br>Includes meat, potato, vegetable meals, no pastry	350g	**Pies[d]**<br>Pastry-containing pies, includes party pies, potato-topped pies (250g)	180g
**Frittata (no pastry)**<br>with Everyday fillings[b]	n/a	**Savoury pastries[d]**<br>Includes sausage rolls, quiche, spring rolls, samosas, pasties	120g
**Savoury scrolls/bread**<br>Includes cheese and spinach, cheese and ham scrolls	n/a	**Cheese and bacon roll or savoury rolls with processed meats[d]**<br>All rolls or pull-apart bread with processed meat	75g
**Mexican meals with soft tortillas or rice**<br>Includes burritos, fajitas, enchiladas	350g	**Mexican food with corn chips or hard taco shells/bowls[d]**<br>Includes hard taco shells or corn chips (30g)	350g
**Pizza (canteen-made)**<br>Canteen-made using only Everyday toppings[b]	350g	**Pizza (packaged)[d] or with Occasional toppings[c]**<br>Packaged pizza any toppings OR canteen-made with Occasional toppings	350g
**Garlic bread/Za'atar/herb bread (canteen–made)**<br>Thin spread of margarine or oil	75g	**Garlic bread/Za'atar bread/herb bread (packaged)[d]**<br>All varieties which are packaged, commercial, ready-to-eat	75g
**Asian meals with rice or noodles**<br>All varieties with rice or noodles (udon/egg/rice)	350g	**Instant flavoured noodles[d]**<br>Includes all flavours	75g dry
**Pasta**<br>Includes lasagne, macaroni and cheese, pasta bakes	350g		
**Indian meals with rice**<br>Includes butter chicken, rogan josh, lentil dahl	350g		
**Dim sum/dumplings/steamed buns (bao)**<br>Steamed or boiled with Everyday fillings[b]	350g		
**Risotto and flavoured rice**<br>with Everyday ingredients[b]	350g		
**Soup**<br>Includes freshly made, ready-to-eat, canned, packet	n/a		
SNACKS – **product categories**		SNACKS – **product categories**	
**Dips – vegetable or yoghurt based**<br>Includes tzatziki, eggplant dips, guacamole, salsa	n/a	**Cheese spread/cream cheese/sour cream-based dips[d]**<br>All cheese/cream cheese-based dips, includes packaged with crackers	60g total
**Plain savoury biscuits/crackers and crispbreads**<br>Includes plain, unsalted crackers, corn and rice cakes	n/a	**Salty snacks[d]**<br>Includes all chips and crisps (potato, corn, grain, vegetable), extruded snacks (cheese, bacon or BBQ flavoured puffed snacks), soy crisps, flavoured or salted rice snacks, flavoured crackers, noodle snacks, pretzels, popcorn (plain, salted or flavoured) and salted legume snacks (salted wasabi peas, roasted fava beans, chickpeas)	30g
**Popcorn (plain, air popped)**<br>Not salted or flavoured	n/a		
**Plain unsalted legume snacks**<br>Includes unsalted roasted chickpeas and fava beans	n/a		
**Scones/pikelets/pancakes/savoury muffins/buns**<br>Includes fruit, cheese or plain scones, fruit buns, hot-cross buns, no icing or confectionary	n/a	**Cakes/donuts/sweet pastries[d]**<br>Includes sweet muffins, banana bread, slices, Danishes, croissants, slices, waffles, oat slices, no icing or confectionary	80g
**Breakfast cereal and muesli[d]**<br>No added confectionary, served with or without milk	n/a	**Muesli bars/snack bars/balls[d]**<br>Includes breakfast bars, protein balls, no yoghurt or confectionery	50g
**Yoghurt and custard**<br>Includes all plain/flavoured, no added confectionary	n/a	**Desserts[d]**<br>Includes mousse, pudding, coconut-milk items, no added confectionary	100g
**Cheese and dairy-free cheese alternatives**<br>Includes colby, tasty, cheddar, ricotta cheese	n/a	**Sweet biscuits[d]**<br>Includes all sweet biscuits, no added confectionary	50g
**Jelly made with ≥99% fruit juice**<br>No added sugar or artificial sweetener	250ml	**Diet jelly**<br>Sweetened with natural or artificial sweeteners (stevia or aspartame)	500g
**Dried fruit**<br>All plain dried fruit with no coating	n/a	**Confectionery[d]**<br>Includes eucalyptus drops, chocolate, lollies, liquorice, carob, yoghurt-type confectionery	50g
**Fruit**<br>All fresh, frozen, canned fruit in juice (not syrup)	n/a		
**Vegetables**<br>All fresh, frozen, canned vegetables	n/a		
DRINKS – **product categories**		DRINKS – **product categories**	
**Water**<br>Includes spring, sparkling, plain water infused with fruit/herb flavouring, no sugar or artificial sweetener	n/a	**Diet drinks and no-sugar drinks**<br>Drinks sweetened with natural or artificial sweeteners such as stevia or aspartame, no added sugar	500ml
**Plain milk and dairy-free milk alternatives**<br>Includes soy, oat, almond milks, full or reduced fat	n/a	**Coffee[d]**<br>All coffee-containing drinks, with/without milk or dairy-free alternatives	500ml
**Flavoured milk/hot chocolate/milkshakes/ smoothies (no ice cream/gelato/sorbet/frozen yoghurt)/breakfast drinks/drinking yoghurt**<br>All milk/dairy free alternatives, no real coffee	500ml	**Milkshakes/thick shakes/smoothies made with icecream/gelato/ sorbet/frozen yoghurt[d]**<br>No more than 1 tablespoon of milk flavouring powder, syrup or honey, no more than 125ml of ice cream/gelato/sorbet	500ml
**≥99% Fruit/vegetable juices and coconut water**	250ml		

Can be diluted with plain or sparkling water, no added sugar or sweeteners			
**FROZEN ICE SNACKS – product categories**		**FROZEN ICE SNACKS – product categories**	
**≥99% juice frozen/blended with ice**<br>Includes ice blocks, frozen drinks, slushies, no added sugar or sweeteners	250ml	**≥99% fruit ice blocks and frozen drinks with added sweeteners**<br>Includes ice blocks, frozen drinks, slushies with natural or artificial sweeteners, no added sugar	500ml
		**Ice cream/gelato/sorbet/frozen yoghurt**[d]<br>Individually packaged, scooped on site, no added confectionery	125ml

[a]Maximum portion size to be classified in this category for secondary schools. Any item exceeding the portion size for Occasional products were classified as 'Should not be sold'.

[b]Everyday fillings/toppings/ingredients include lean meats, tuna, salmon, prawns, other seafood, egg, cheese, ham, salad/vegetables/roast vegetables, fruit, un-coated falafel, avocado, hummus, un-coated tofu, rice noodles, legumes, wholegrains, baked beans, tinned spaghetti. Lean meats can include skinless chicken, steak, roast beef, turkey breast, low fat mince, lamb and pork.

[c]Occasional fillings/toppings/ingredients include processed meat (see table) and packaged crumbed/coated foods (see table), battered or tempura foods

[d]Minimum 3.5 Health Star Rating (HSR) for packaged, commercial, ready-to-eat products. Only one Everyday product category (Breakfast cereal and museli) requires a 3.5 HSR. More information regarding HSR can be found at http://www.healthstarrating.gov.au
